# Phylogeny and Taxonomic Revision of the Family *Discinaceae* (*Pezizales*, *Ascomycota*)

**DOI:** 10.1128/spectrum.00207-23

**Published:** 2023-04-27

**Authors:** Xin-Cun Wang, Zhu-Liang Yang, Shuang-Lin Chen, Tolgor Bau, Tai-Hui Li, Lin Li, Li Fan, Wen-Ying Zhuang

**Affiliations:** a State Key Laboratory of Mycology, Institute of Microbiology, Chinese Academy of Sciences, Beijing, China; b Key Laboratory for Plant Diversity and Biogeography of East Asia, Kunming Institute of Botany, Chinese Academy of Sciences, Kunming, China; c College of Life Sciences, Nanjing Normal University, Nanjing, China; d Key Laboratory of Edible Fungi Resources and Utilization (North), Ministry of Agriculture and Rural Affairs, Jilin Agricultural University, Changchun, Jilin, China; e Guangdong Provincial Key Laboratory of Microbial Culture Collection and Application, State Key Laboratory of Applied Microbiology Southern China, Guangdong Institute of Microbiology, Guangdong Academy of Sciences, Guangzhou, China; f College of Agronomy and Biosciences, Dali University, Dali, China; g College of Life Science, Capital Normal University, Beijing, China; Chinese Academy of Sciences

**Keywords:** *Ascomycota*, phylogeny, taxonomy, 3 new genera, 2 new species

## Abstract

Species of *Discinaceae* are common macrofungi with a worldwide distribution. Some of them are commercially consumed, while a few others are reported as poisonous. Two genera were accepted in the family: the epigeous Gyromitra with discoid, cerebriform to saddle-shaped ascomata and the hypogeous Hydnotrya with globose or tuberous ascomata. However, due to discrepancies in their ecological behaviors, a comprehensive investigation of their relationship was not thoroughly explored. In this study, phylogenies of *Discinaceae* were reconstructed using sequence analyses of combined and separate three gene partitions (internal transcribed spacer [ITS], large subunit ribosomal DNA [LSU], and translation elongation factor [TEF]) with a matrix containing 116 samples. As a result, the taxonomy of the family was renewed. Eight genera were recognized: two of them (Gyromitra and Hydnotrya) were retained, three (Discina, Paradiscina, and Pseudorhizina) were revived, and three (Paragyromitra, Pseudodiscina, and Pseudoverpa) were newly established. Nine new combinations were made in four genera. Two new species in Paragyromitra and Pseudodiscina and an un-named taxon of Discina were described and illustrated in detail based on the materials collected from China. Furthermore, a key to the genera of the family was also provided.

**IMPORTANCE** Taxonomy of the fungal family *Discinaceae* (*Pezizales*, *Ascomycota*) was significantly renewed on the basis of sequence analyses of internal transcribed spacer (ITS), large subunit ribosomal DNA (LSU), and translation elongation factor (TEF). Eight genera were accepted, including three new genera; two new species were described; and nine new combinations were made. A key to the accepted genera of the family is provided. The aim of this study is to deepen the understanding of the phylogenetic relationships among genera of the group, as well as the associated generic concepts.

## INTRODUCTION

Species of *Discinaceae* Benedix are epigeous or hypogeous macrofungi having discoid, cerebriform, saddle-shaped, or tuberous ascomata. They are distributed worldwide; for example, Discina melaleuca Bres. is found in Europe (France and Germany), Gyromitra xinjiangensis J.Z. Cao et al. is in Asia (China), Pseudorhizina californica (W. Phillips) Harmaja occurs in North America (Canada and USA), Gyromitra antarctica Rehm grows in South America (Argentina and Chile), and Gyromitra tasmanica Berk. & Cooke is reported from Oceania (Australia and New Zealand). A few species are commercially consumed; e.g., Gyromitra esculenta (Pers.) Fr. is commonly sold and eaten in Estonia, Finland, Poland, and Sweden. Gyromitra esculenta and Gyromitra gigas (Krombh.) Cooke were evaluated as “edible with caution” in a recent review (1). Poisonous gyromitrins are well known from G. esculenta, G. gigas, Gyromitra ambigua (P. Karst.) Harmaja, and Gyromitra infula (Schaeff.) Quél., which cause a series of symptoms: vomiting, bloody diarrhea, abdominal pain, dehydration, sweating, headache, myalgias, vertigo, delirium, hemolysis, hepatic damage, kidney failure, and so forth (2, 3).

In 1753, Linnaeus established the genus Helvella (as “*Elvela*”) for Helvella mitra L. Then Schaeffer added Helvella infula to the genus in 1774. Persoon changed the latter name to Helvella esculenta according to the priority rule in 1800 (4). In 1822, Fries classified Discina as a subgenus of Peziza Dill. ex Fr. to accommodate *P. perlata* Fr. Later in 1849, he promoted Discina to a full genus with only one species, also known as a monotypic genus (5). In the same protologue, Fries established a separate genus, Gyromitra Fr., which also only contained one species, G. esculenta.

The family *Helvellaceae* (as “*Elvellaceae*”) was established by Fries in 1822 to include almost all operculate cup fungi (6). In 1907, Boudier erected the tribe *Discineae* in *Pezizaceae* and placed Gyromitra in *Helvellaceae* (7). In 1961, Benedix proposed a separate family *Discinaceae* to include Discina, Maublancomyces Herter (typified with Helvella gigas Krombh.) and Neogyromitra S. Imai (typified by Neogyromitra caroliniana (Bosc) S. Imai); and placed Gyromitra and Pseudorhizina Jacz. in *Helvellaceae* (8, 9). Dissing did not accept *Discinaceae* but remained the tribe *Discineae* along with the tribes *Gyromitreae* and *Helvelleae* in *Helvellaceae* (10). Eckblad affiliated Discina, Gyromitra and Pseudorhizina with *Rhizinaceae* (11), whereas Discina, Neogyromitra, Paradiscina Benedix, and Pseudorhizina were synonymized with Gyromitra by Harmaja (12, 13). Trappe moved the hypogeous Hydnotrya Berk. and Broome to *Helvellaceae* (14). Thus, *Helvellaceae*, in a broad sense, included Gyromitra, Helvella, Pseudorhizina, Rhizina Fr., Underwoodia Peck and the hypogeous genera, Balsamia Vittad., Barssia Gilkey, Hydnotrya, etc., which was followed by some other taxonomists (15–17).

The above taxonomic treatments were challenged by molecular systematics initially based on nuclear ribosomal DNA in the 1990s. Discina, Gyromitra, Hydnotrya, and Pseudorhizina turned out to be forming a monophyletic clade, which was distinct from the clade containing Balsamia, Barssia, Helvella, Underwoodia, and Wynnella Boud. This result indicated that both *Helvellaceae* and *Discinaceae* should be separated at the family level (18), which was supported by the subsequent phylogenetic studies (19–21). The introduction of additional sequences of large subunit ribosomal DNA (LSU) from more *Discinaceae* taxa resulted in the emergence of divisions within Gyromitra. Specifically, Discina and Pseudorhizina were reclassified as subgenera of Gyromitra (22). Similar results were showed by the multigene analyses inferred from LSU, internal transcribed spacer (ITS), and translation elongation factor 1-α (TEF1-α) genes; however, subgen. Discina was not monophyletic, and thus, Gyromitra in a narrow sense was suggested (23).

The phylogeny of *Discinaceae* was reconstructed based on three individual and combined gene partitions (ITS, LSU, and TEF) in this study. Three new genera were proposed, two new species were described and illustrated, and nine new combinations were made based on the combined molecular and morphological data.

## RESULTS

A total of 92 samples from *Discinaceae* and 24 samples from related families, namely, *Geomoriaceae*, *Helvellaceae*, *Morchellaceae*, and *Tuberaceae*, were examined for their sequences. The details, e.g., alignment length, numbers of variable, and informative sites of these data sets, are given in Table 1.

**TABLE 1:** Detailed characteristics of data sets of *Discinaceae*^*a*^

Locus	No. of sequences	Length of alignment (bp)	No. of variable sites	No. of parsimony-informative sites	Model for BI
ITS+LSU+TEF	85	3,279	1,790	1,412	GTR+I+G
ITS	90	1,898	1,313	977	
LSU	92	875	294	228	
TEF	58	587	279	254	

a GTR+I+G, general time reversible with invariable sites and gamma distribution; ITS, internal transcribed spacer; LSU, large subunit ribosomal DNA; TEF, translation elongation factor.

**FIG 1:**
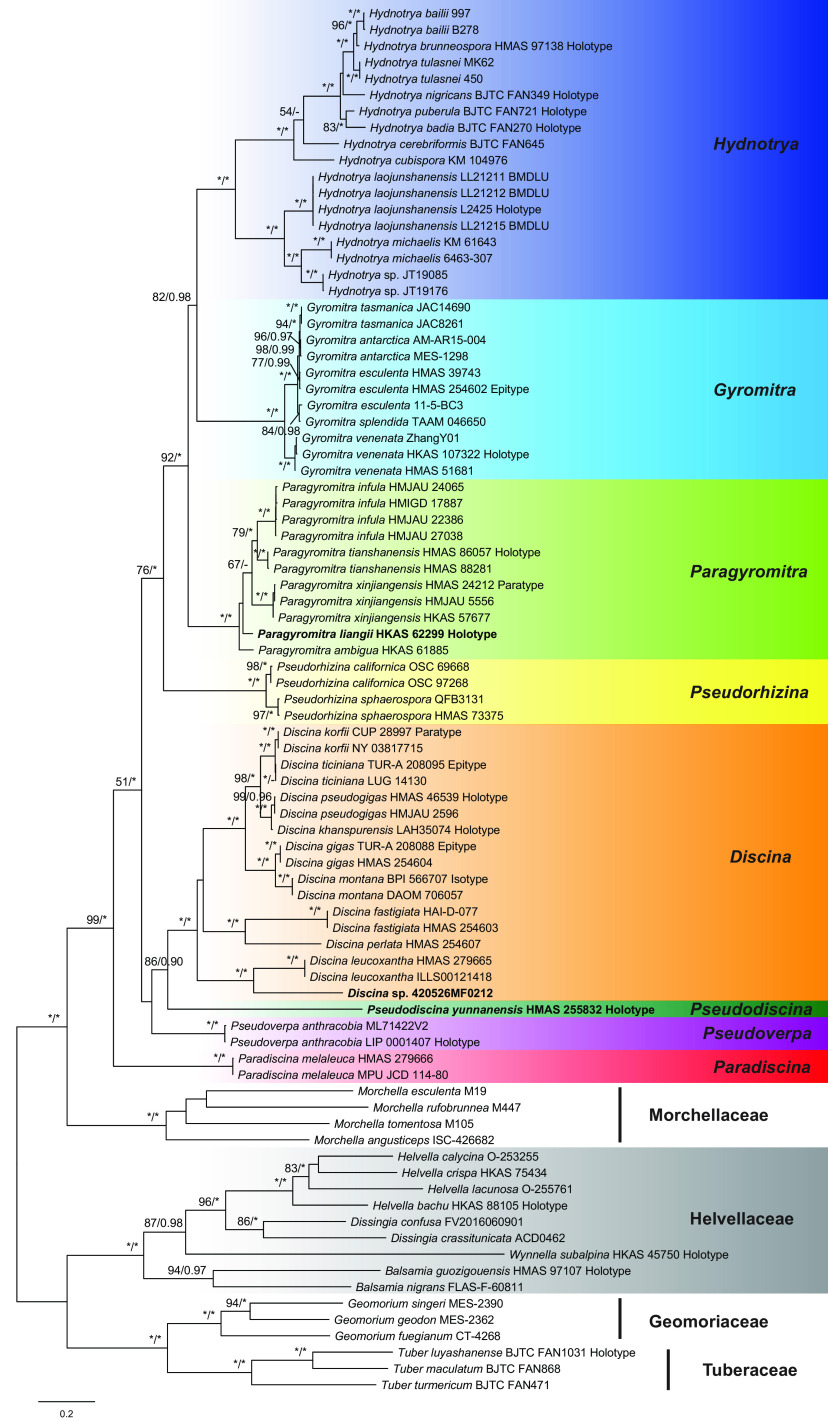
Maximum-likelihood phylogenetic tree of *Discinaceae* inferred from combined internal transcribed spacer (ITS), large subunit ribosomal DNA (LSU), and translation elongation factor 1 (TEF1) data sets. Maximum-likelihood bootstrap percentage (MLBP) ≥ 50% (left), and Bayesian inference posterior probability (BIPP) ≥ 0.90 (right) are indicated at nodes. Asterisks denote 100% bootstrap values or 1.00 posterior probability values.

The 3-locus data set contained 85 taxa in an alignment including 1,827 bp of ITS, 870 bp of LSU, and 582 bp of TEF. The general time reversible model with invariable sites and gamma distribution (GTR+I+G) was selected by Akaike information criterion as the best fit for Bayesian inference analysis. The phylogeny of the family *Discinaceae* was reconstructed, and the monophyly of the family was determined and illustrated in Fig. 1. Eight clades corresponding to eight genera were recognized and are strongly supported with 100% maximum-likelihood bootstrap percentage (MLBP) values and 1.00 Bayesian inference posterior probability (BIPP). Paradiscina was positioned as the foundational lineage of the tree. A sister relationship between Discina and Pseudodiscina was supported by both maximum-likelihood (ML) and Bayesian inference (BI) analyses (86% MLBP, 0.90 BIPP). The remaining four genera formed a well supported group (76% MLBP, 1.00 BIPP), including Hydnotrya (hypogeous), Gyromitra (epigeous), Paragyromitra (epigeous), and Pseudorhizina (epigeous). Sister relationship between Hydnotrya and Gyromitra was confirmed (100% MLBP, 1.00 BIPP). Three undescribed species appeared in three genera. The collection 420526MF0212 (Discina sp.) was a sister of Discina leucoxantha, HKAS 62299 (Paragyromitra sp.) was a sister of Paragyromitra ambigua, and HMAS 255832 (= Yang 6275) was the representative of Pseudodiscina closely related to Discina.

**FIG 2:**
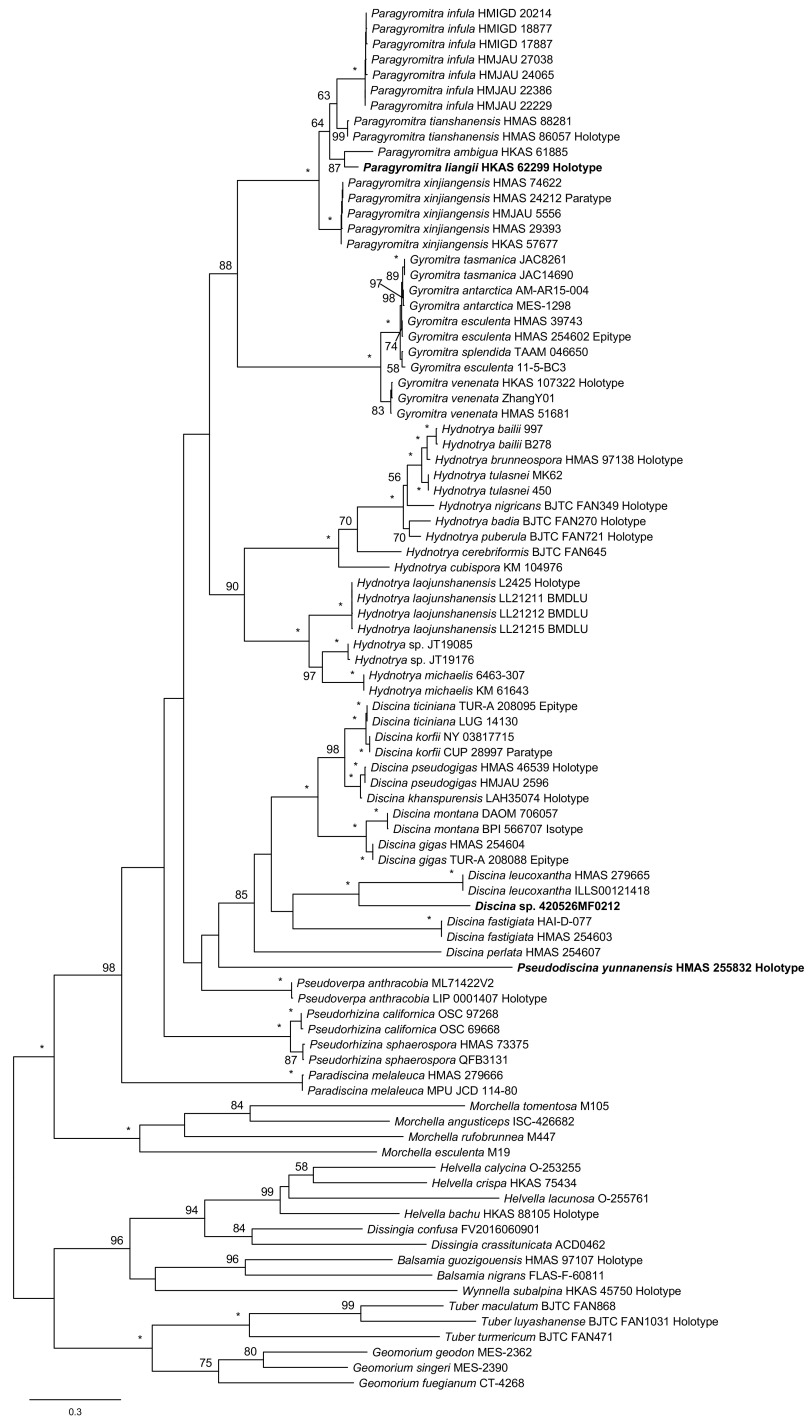
Maximum-likelihood phylogenetic tree of *Discinaceae* inferred from the ITS data set. MLBP ≥ 50% are indicated at nodes. Asterisks denote 100% bootstrap values.

In Fig. 2, which represents the phylogeny of the ITS sequences, the eight genera were distinctly separated. Both Paradiscina and Pseudorhizina were positioned at the base of the tree. The analysis indicated a close relationship between Gyromitra and Paragyromitra (88% MLBP).

**FIG 3:**
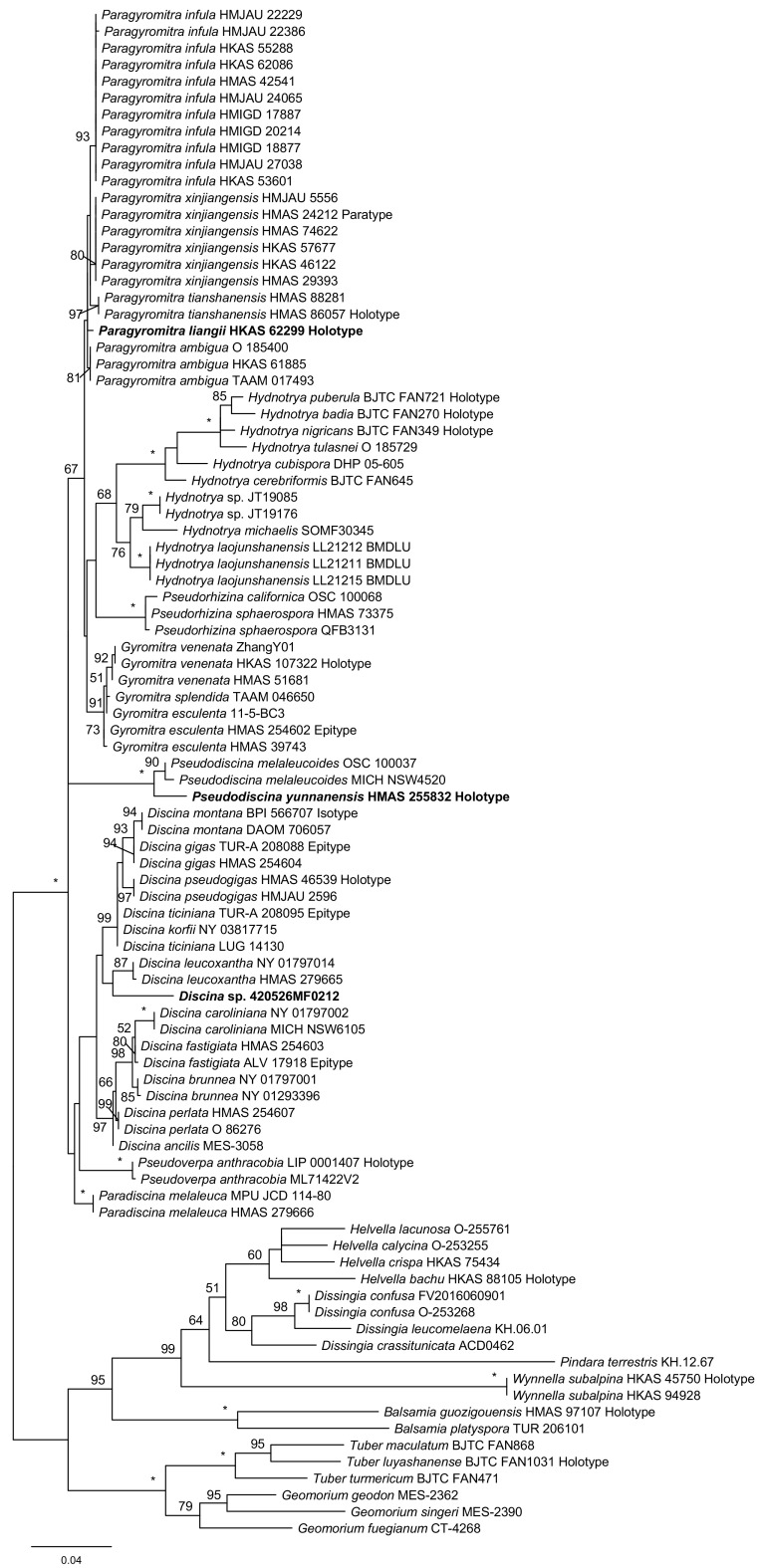
Maximum-likelihood phylogenetic tree of *Discinaceae* inferred from the LSU data set. MLBP ≥ 50% are indicated at nodes. Asterisks denote 100% bootstrap values.

In the LSU phylogeny (Fig. 3), the eight genera formed eight clades. The close relationships among Hydnotrya, Gyromitra, Paragyromitra, and Pseudorhizina was again supported. HMAS 255832 was sister to Pseudodiscina melaleucoides (100% MLBP).

**FIG 4:**
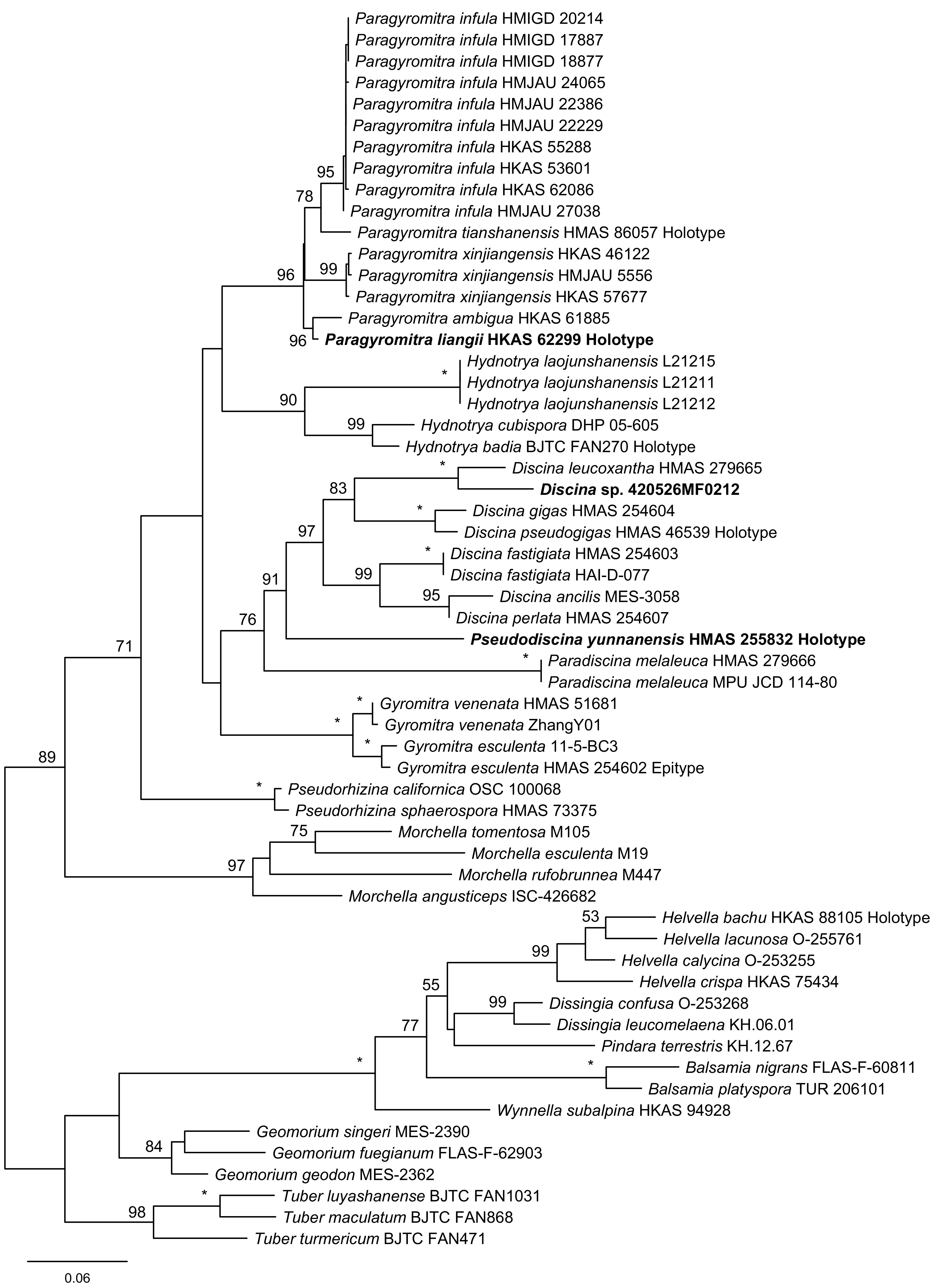
Maximum-likelihood phylogenetic tree of *Discinaceae* inferred from the TEF data set. MLBP ≥ 50% are indicated at nodes. Asterisks denote 100% bootstrap values.

In the TEF phylogeny, seven of the eight genera were included and well differentiated, as shown in Fig. 4. The basal clade was represented by Pseudorhizina. The close relationships among Discina, Pseudodiscina, and Paradiscina were confirmed. The phylogenetic positions of the three undescribed species were obviously distinguishable.

Compared with the topologies of the single-gene trees, the three-locus phylogeny strongly supported the sister relationship between Gyromitra and Hydnotrya and the close association of Pseudorhizina to Gyromitra, Hydnotrya, and Paragyromitra. The sister relationships between Discina and Pseudodiscina were not supported by the ITS or LSU analyses but were confirmed by the three-locus and TEF phylogenies. Although Paradiscina shown as a basal clade was revealed by the ITS and LSU trees, the early divergence of this genus was clearly demonstrated by the three-locus analysis.

## TAXONOMY

***Discinaceae* Benedix**, Z. Pilzk. 27(2–4): 100, 1962.

= *Pseudorhizinaceae* Harmaja, Karstenia 14: 14, 1974.

Type genus: Discina (Fr.) Fr., Summa veg. Scand., Sectio Post.: 348, 1849.

Ascomata epigeous or hypogeous; discoid, cupulate, cerebriform, or campanulate; subsessile to stipitate in apothecial forms; tuberculate or subglobose in ptychothecial forms. Excipulum of textura intricata or poorly defined textura angularis in apothecial forms; excipulum/peridium stratified in ptychothecial forms. Paraphyses filiform, forming epithecium in some ptychothecial taxa. Asci inamyloid, operculate in apothecial taxa, indehiscent in ptychothecial taxa; typically with eight spores maturing; sporogenous region subcylindric to clavate. Ascospores ellipsoidal, broadly ellipsoidal, globose, fusoidal, or rectangular; apiculate or nonapiculate; smooth, roughened, echinate, or warted; uni-, bi-, or triguttulate in apothecial taxa; guttules sometimes visible when young but obscured at maturity in hypogeous taxa.

Notes: Members of *Discinaceae* had been placed in three families, e.g., Gyromitra in *Helvellaceae* (10) or in *Rhizinaceae* (12) and Pseudorhizina in *Pseudorhizinaceae* (24). With the aid of molecular systematics, the families became well established. In *Pezizales*, *Discinaceae* consisting of eight accepted genera has been shown as sister family of *Morchellaceae* (Fig. 1). Most of the accepted genera are epigeous, including Discina, Gyromitra, Paradiscina, Paragyromitra, Pseudodiscina, Pseudorhizina, and Pseudoverpa except for the hypogeous Hydnotrya. A revised classification of the family is proposed.

Key to genera of *Discinaceae*

1. Ascomata hypogeous… … … … … … … … … … … … … … ……Hydnotrya

1. Ascomata epigeous… … … … … … … … … … … … … … … … … …2

2. Ascospores apiculate, triguttulate or uniguttulate… … … … … … … … … Discina

2. Ascospores not apiculate, biguttulate… … … … … … … … … … … … … …3

3. Apothecia discoid to cupulate… … … … … … … … … … … … … … … …4

3. Apothecia other shaped. … … … … … … … … … … … … … … … … … …5

4. Apothecial extreme margin slightly elevated… … … … … … … … … …Paradiscina

4. Apothecial extreme margin reflexed… … … … … … … … … …Pseudodiscina

5. Apothecia usually saddle-shaped… … … … … … … … … … … …Paragyromitra

5. Apothecia cerebriform or irregularly lobed… … … … … … … … … … … … …6

6. Stipe deeply ribbed… … … … … … … … … … … … … … … …Pseudorhizina

6. Stipe subcylindrical… … … … … … … … … … … … … … … … … … …7

7. Apothecia cerebriform … … … … … … … … … … … … … … …Gyromitra

7. Apothecia somewhat campanulate… … … … … … … … … … …Pseudoverpa

**Discina** (Fr.) Fr., Summa veg. Scand., Sectio Post.: 348, 1849.

≡ Peziza a Discina Fr., Syst. Mycol. 2 (1): 38, 1822.

= Neogyromitra S. Imai, Bot. Mag. 46: 174, 1932 (Type species: Discina caroliniana (Bosc) Eckblad, Nytt Mag. Bot. 15[1–2]: 100, 1968).

= Maublancomyces Herter, Revista Südam. de Botanica 8: 161, 1950 (Type species: Discina gigas (Krombh.) Eckblad, Nytt Mag. Bot. 15[1–2]: 99, 1968).

= Fastigiella Benedix, Kulturpflanze 17: 276, 1969 (Type species: D. caroliniana (Bosc) Eckblad).

Type species: Discina perlata (Fr.) Fr., Summa veg. Scand., Sectio Post.: 348, 1849.

≡ Peziza perlata Fr., Syst. Mycol. 2(1): 43, 1822.

≡ Gyromitra perlata (Fr.) Harmaja, Karstenia 9: 11, 1969.

Ascomata discoid, cupulate to lobed, sessile to stipitate. Hymenium yellow brown, orange brown, reddish brown, to dark brown; surface undulate-rugose to convoluted-wrinkled. Asci operculate, mostly eight-spored, subcylindrical. Ascospores ellipsoidal to fusoidal, uniguttulate or triguttulate, distinctly roughened to reticulate, with or without solitary or multiple polar apiculi.

Notes: This genus was previously treated as a subgenus of Gyromitra by Harmaja (25), which was followed by the subsequent studies (15, 22, 23). Based on our analyses, Discina includes the previously treated Gyromitra subgen. caroliniana S.P. Abbott and Gyromitra subgen. Discina (Fr.) Harmaja sensu S.P. Abbott (15). The genus is monophyletic (Fig. 1 to 4) and associated with Pseudodiscina (Fig. 1 and 4). Thirteen species are currently known in the genus.

**Discina khanspurensis** (Jabeen and Khalid) X.C. Wang and W.Y. Zhuang, comb. nov.

≡ Gyromitra khanspurensis Jabeen and Khalid, Sydowia 69: 236, 2017.

Fungal Names FN571026

Notes: Our phylogenetic analysis (Fig. 1) showed that this species belongs to Discina rather than Gyromitra. Originally described from Pakistan (26), this species is closely related to Gyromitra pseudogigas but can be distinguished from it by having smaller and wider ascospores and narrower asci (23, 27).

**Discina pseudogigas** (X.C. Wang & W.Y. Zhuang) X.C. Wang & W.Y. Zhuang, comb. nov.

≡ Gyromitra pseudogigas X.C. Wang & W.Y. Zhuang, Mycologia 111(1): 73, 2019.

Fungal Names FN571027

Specimen examined: China, Heilongjiang Province, Mudanjiang City, Hailin City, on ground of forest, 9 August 2004, Tolgor Bau, HMJAU 2596.

Notes: The apothecia of the fungus from Heilongjiang (HMJAU 2596) are discoid rather than saddle-shaped as the holotype HMAS 46539 from Sichuan (23). Variation in apothecial shape is considered infraspecific variation.

**Discina ticiniana** (Littini) X.C. Wang & W.Y. Zhuang, comb. nov.

≡ Gyromitra ticiniana Littini, Pagine Botaniche 12: 19, 1988.

= Gyromitra littiniana A. Riva, Schweiz. Z. Pilzk. 88(6): 233, 2010.

Fungal Names FN571028

Notes: The close relationship between Discina korfii and Discina ticiniana was confirmed (Fig. 1 to 3). Detailed description and taxonomic treatment of the fungus were provided by Carbone et al. (28).

**FIG 5:**
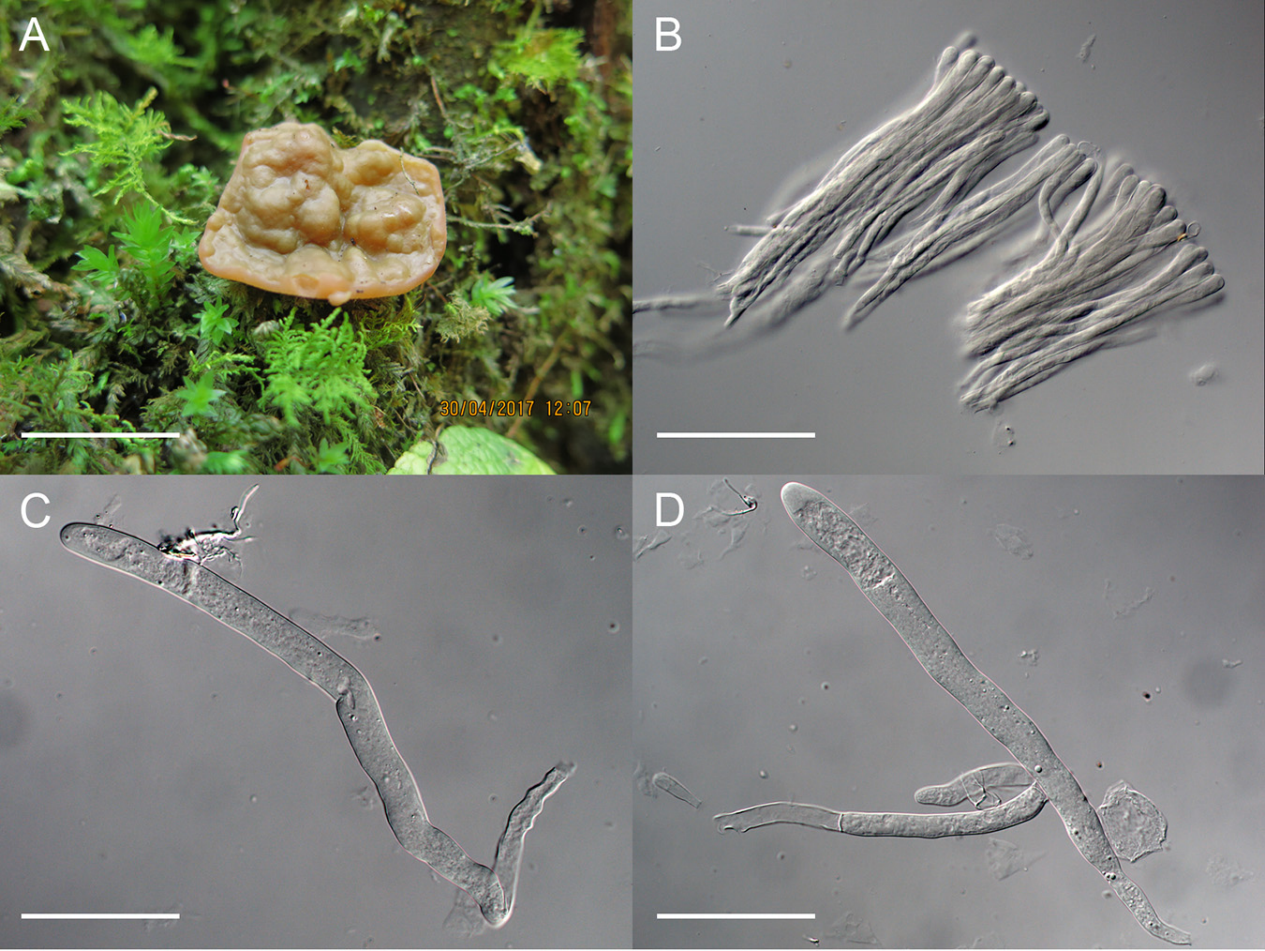
Discina sp. 420526MF0212. (A) Fresh apothecium on nature substrate. (B) Paraphyses. (C, D) Asci. Bars = 2 cm (A), 70 μm (B), 70 μm (C), and 85 μm (D).

**Discina** sp. 420526MF0212 (Fig. 5)

Specimen examined: China, Hubei Province, Yichang City, Xingshan County, Longmenhe National Forest Park, 31°18′24″N, 110°28′28″E, Alt. 1572 m, on ground of forest, 30 April 2017, Xian Zhang, 420526MF0212 (previously filed under Gyromitra leucoxantha).

Apothecia discoid, sessile to substipitate, up to 3 cm in diameter when fresh, 1.5 cm in diameter and 1 cm high when dry; hymenium yellow brown when fresh, dark brown to blackish when dry, undulate-rugose; receptacle surface whitish, glabrous. Excipulum of textura intricata, hyphae hyaline, 10 to 16 μm wide. Asci not fully mature, subcylindrical, tapering toward base, absence of ascospores, 315 to 330 × 13 to 21 μm. Paraphyses filiform, bifurcate, septate, hyaline, 8 to 10.5 μm wide at apex, 5.5 to 6.5 μm below.

Notes: Phylogenetically, Discina sp. 420526MF0212 was a sister of Discina leucoxantha in the phylogenetic trees based on the ITS, TEF, and multilocus sequences (Fig. 1 to 4). It differs from the latter in blackish hymenium when dry instead of bright red brown (15), in addition to the sequence data. Apothecium of the fungus is immature, and ascospore was not observed in the asci. Among the known species of the genus lacking of molecular data, Discina accumbens Rahm differs in reddish brown hymenium when fresh; Discina geogenia E. Rahm ex Donadini is of reddish brown to blackish hymenium when fresh; Discina martinii (Donadini & Astier) Donadini & Astier has dark brown hymenium when fresh; and Discina parma J. Breitenb. & Maas Geest. possesses reddish brown hymenium when fresh (data from Ascomycete.org, https://ascomycete.org/). The sequence data indicated that it might represent an undescribed species, waiting for future collections with mature ascospores.

**Gyromitra** Fr., Summa veg. Scand., Sectio Post.: 346, 1849.

= Physomitra Boud., Bull. Soc. Mycol. Fr. 1: 99, 1885.

Type species: G. esculenta (Pers.) Fr., Summa Veg. Scand., Sectio Post.: 346, 1849.

Ascomata cerebriform, irregularly lobed, stipitate; stipe subcylindrical, internally hollow. Hymenium yellow brown, reddish brown, to dark brown. Asci operculate, eight-spored, subcylindrical. Ascospores narrowly ellipsoidal, ellipsoidal, or fusoidal; nonapiculate, hyaline, smooth, biguttulate, irregularly biseriate when young, uniseriate at maturity.

Notes: The genus is difficult to distinguish from Paragyromitra in gross morphology. Gyromitra clustered with Hydnotrya and Paragyromitra in our phylogenetic trees (Fig. 1 to 3). To date, G. antarctica and G. tasmanica are the only species of the family that occurred in the Southern Hemisphere. Five species are currently known, and Gyromitra venenata Hai J. Li et al. is the only one found in China (29).

**Hydnotrya** Berk. & Broome, Ann. Mag. Nat. Hist., Ser. 1, 18: 78, 1846.

Type species: Hydnotrya tulasnei (Berk.) Berk. & Broome, Ann. Mag. Nat. Hist., Ser. 1, 18: 78, 1846.

≡ Hydnobolites tulasnei Berk., Ann. Mag. Nat. Hist., Ser. 1, 13: 357, 1844.

Ascomata hypogeous, irregularly subspherical, ellipsoidal to tuberculate, compact or single-chambered, surface wrinkled. Asci eight-spored, subcylindrical. Ascospores globose, broadly ellipsoidal to rectangular, nonapiculate, honey-yellow, reddish orange to brown, with a large central guttule, thick-walled, nodulose-verrucose to echinulate, with rounded or truncate apices.

Notes: Abbott (15) recognized Hydnotrya with two subgenera by establishing Hydnotrya subgen. cerebriformes based on H. cerebriformes Harkn. because of its echinate ascospores. However, our phylogenetic analyses did not support his taxonomic treatment (Fig. 1 to 3), in which the genus is a sister to Gyromitra and Paragyromitra. Fourteen species are recognized in the genus (30), and six of them have been reported from China.

**Paradiscina Benedix**, Kulturpflanze 17: 274, 1969.

Type species: Paradiscina melaleuca (Bres.) Benedix, Kulturpflanze 17: 275, 1969.

≡ Discina melaleuca Bres., Fung. Trident. 2(11–13): 74, 1892.

≡ Pachyella melaleuca (Bres.) Boud., Hist. Class. Discom. Eur.: 50, 1907.

≡ Peziza melaleuca (Bres.) Seaver, North American cup fungi (Operculates): 225, 1928.

≡ Gyromitra melaleuca (Bres.) Donadini, Bull. Soc. Linn. Provence 28: 74, 1976.

Ascomata cupulate or discoid, sessile or substipitate. Asci operculate, eight-spored, subcylindrical. Ascospores ellipsoidal, nonapiculate, biguttulate, warted to coarsely rugose.

Notes: After Benedix (31) established the genus Paradiscina, seven additional species were introduced (31, 32). With the exception of its type species, all other species are considered to be members of Discina. The genus at the current sense is therefore monotypic. Its association with Discina and Pseudodiscina was supported by the TEF phylogeny (Fig. 4), whereas its location at the base of the *Discinaceae* tree was revealed in the three-locus, ITS, and LSU analyses (Fig. 1 to 3).

**Paragyromitra** X.C. Wang & W.Y. Zhuang, gen. nov.

Fungal Names FN571031

Etymology: The generic name refers to its resemblance to Gyromitra in gross morphology.

Type species: G. infula (Schaeff.) Quél.

Ascomata saddle-shaped, cerebriform or irregularly lobed, stipitate; stipe subcylindrical, internally hollow. Asci operculate, eight-spored, subcylindrical. Ascospores narrowly ellipsoidal, ellipsoidal or fusoidal, hyaline, smooth, biguttulate, nonapiculate.

Notes: The new genus is a separation from Gyromitra and along with recognition of the hypogeous Hydnotrya (Fig. 1 to 4). Morphologically, the genus is very similar to Gyromitra but mostly having saddle-shaped ascomata. Five species are known in the genus, which all occur in China.

**Paragyromitra ambigua** (P. Karst.) X.C. Wang & W.Y. Zhuang, comb. nov.

≡ Helvella ambigua P. Karst., Meddn Soc. Fauna Flora Fenn. 5: 53, 1879.

≡ Gyromitra ambigua (P. Karst.) Harmaja, Karstenia 9: 17, 1969.

= Gyromitra infula var. *apiculatispora* Raitv., Eesti NSV Tead. Akad. Toim., Biol. Seer 14(2): 322, 1965.

Fungal Names FN571032

Specimen examined: China, Heilongjiang Province, Daxing’anling Prefecture, Huzhong District, Huzhong National Reserve, Dabai Mountain, 51°37’19″N, 123°31’47″E, 22 August 2010, Xiang-Hua Wang 2688, HKAS 61885 (previously filed under Gyromitra sp.).

Notes: This species was originally described from Finland. Its distribution in China was first reported from Jilin Province (33). Sequence analysis suggests its placement in Paragyromitra.

**Paragyromitra infula** (Schaeff.) X.C. Wang & W.Y. Zhuang, comb. nov.

≡ Helvella infula Schaeff., Fung. Bavar. Palat. Nasc. 4: 105, 1774.

≡ Gyromitra infula (Schaeff.) Quél., Enchir. Fung.: 272, 1886.

≡ Physomitra infula (Schaeff.) Boud., Hist. Class. Discom. Eur.: 35, 1907.

Fungal Names FN571033

Notes: This species is widely distributed in the Northern Hemisphere. It had been reported from Asia (China), Europe (France), and North America (Canada, Mexico, and USA) (22, 23). Its occurrence in Belarus (HMJAU 22229 and HMJAU 22386) was confirmed by this study. It was recorded from Sichuan, Tibet, Xinjiang, and Yunnan in southwestern and northwestern China (23) and now extends to Hubei, Inner Mongolia, and Jilin provinces.

**FIG 6:**
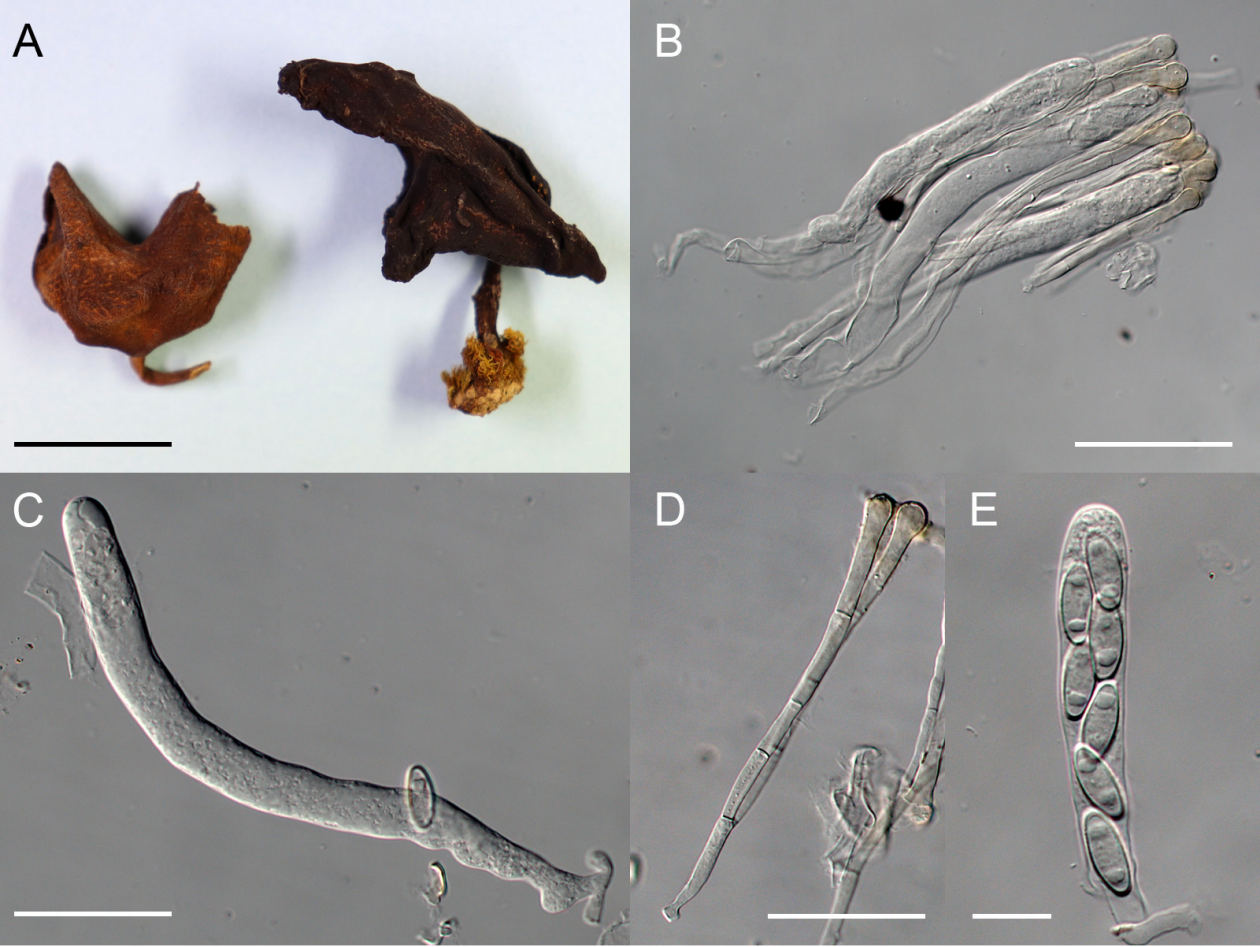
Paragyromitra liangii (HKAS 62299). (A) Dried apothecia. (B) Asci and paraphyses. (C) Young ascus. (D) Paraphyses. (E) Ascospores in ascus. Bars = 1.5 cm (A), 70 μm (B), 55 μm (C, D), and 20 μm (E).

**Paragyromitra liangii** X.C. Wang & W.Y. Zhuang, sp. nov. (Fig. 6).

Fungal Names FN571034

Etymology: The specific epithet is in memory of the late distinguished mycologist Zong-Qi Liang.

Typification: China, Yunnan Province, Diqing Tibetan Autonomous Prefecture, Shangri-La City, at south line of Bitahai Lake, 27°49'21″N, 99°59'52″E, 15 September 2010, on ground, Xiao-Fei Shi, HBB2010-SH-104, HKAS 62299, holotype (previously filed under Gyromitra sp.).

Apothecia saddle-shaped or irregularly lobed, stipitate, 1.9 to 3.3 cm in diameter and 3 to 3.5 cm high when dry; hymenium dull reddish brown, dark brown to blackish when dry, undulate-rugose; receptacle surface light brown to blackish, glabrous; stipe subcylindrical, slightly enlarged at base, light brown to blackish, glabrous, internally hollow, 1.5 × 0.1 to 0.3 cm. Excipulum of textura intricata, hyphae hyaline, 7.5 to 17.5 μm wide. Asci subcylindrical, tapering at base, eight-spored, 185 to 250 × 14.5 to 22.5 μm. Ascospores narrowly ellipsoidal to fusoidal, hyaline, smooth to finely roughened, biguttulate, nonapiculate, irregularly biseriate when young, uniseriate at maturity, 20 to 25 × 7.5 to 9 μm. Paraphyses filiform to clavate, bifurcate, septate, hyaline but light brown at apex, 8 to 12 μm wide at apex and 4 to 5 μm below.

Notes: Phylogenetically, Paragyromitra liangii was sister to P. ambigua in the phylogenies based on the multilocus, ITS, and TEF sequence analyses (Fig. 1 to 4). It differs from the latter in 687 of 1367 bp for ITS, 4 of 870 bp for LSU, and 13 of 559 bp for TEF. Its apothecia possess very thin stipes compared with other known species of the genus (15).

**Paragyromitra tianshanensis** (X.C. Wang & W.Y. Zhuang) X.C. Wang & W.Y. Zhuang, comb. nov.

≡ Gyromitra tianshanensis X.C. Wang & W.Y. Zhuang, Mycologia 111(1): 72, 2019.

Fungal Names FN571035

Notes: This species was originally described from Xinjiang Uygur Autonomous Region of China (23), and its occurrence in South Korea was recently reported (34).

**Paragyromitra xinjiangensis** (J.Z. Cao, L. Fan & B. Liu) X.C. Wang & W.Y. Zhuang, comb. nov.

≡ Gyromitra xinjiangensis J.Z. Cao, L. Fan & B. Liu, Acta Mycol. Sin. 9(2): 105, 1990.

Fungal Names FN571036

Notes: This species has a broad distribution in China, and was found in Gansu, Jilin, Sichuan, Tibet, Xinjiang (23), and Yunnan. To date, there have not been reports of this species outside China.

**Pseudodiscina** X.C. Wang & W.Y. Zhuang, gen. nov.

Fungal Names FN571037

Etymology: The generic name refers to its resemblance to the genus Discina.

Type species: Gyromitra melaleucoides (Seaver) Pfister.

Ascomata cupulate or discoid, stipitate; hymenium dark brown to blackish when dry; receptacle surface whitish, buff to yellow brown when dry, glabrous to finely pubescent; stipe subcylindrical, whitish, yellow brown to light brown, glabrous to finely pubescent, internally hollow. Asci operculate, eight-spored, subcylindrical. Ascospores ellipsoidal to broadly ellipsoidal, nonapiculate, biguttulate, with rough surface.

Notes: Abbott and Currah (15) established Gyromitra subgen. melaleucoides to accommodate G. melaleucoides and G. melaleuca, but the monophyly of the subgenus was not supported by the molecular data, and G. melaleucoides represents a separate genus (Fig. 1 to 4). In the multilocus tree (Fig. 1), Pseudodiscina was clustered with Discina and Pseudoverpa, and it was a sister of Discina and Paradiscina in the TEF inference (Fig. 4). Two species of Pseudodiscina were accepted, including a new one.

**Pseudodiscina melaleucoides** (Seaver) X.C. Wang & W.Y. Zhuang, comb. nov.

≡ Peziza melaleucoides Seaver, North American cup fungi (Operculates): 225, 1928.

≡ Gyromitra melaleucoides (Seaver) Pfister, Mycologia 72(3): 615, 1980.

= Gyromitra recurva (Snyder) Harmaja, Karstenia 18(2): 57, 1978.

≡ Paxina recurvum Snyder, Mycologia 28(5): 487, 1936.

Fungal Names FN571038

Notes: This species is possibly endemic to the Rocky Mountains in North America, including in Alberta and British Columbia in Canada and Alaska, Colorado, Idaho, and Washington in the USA (15).

**FIG 7:**
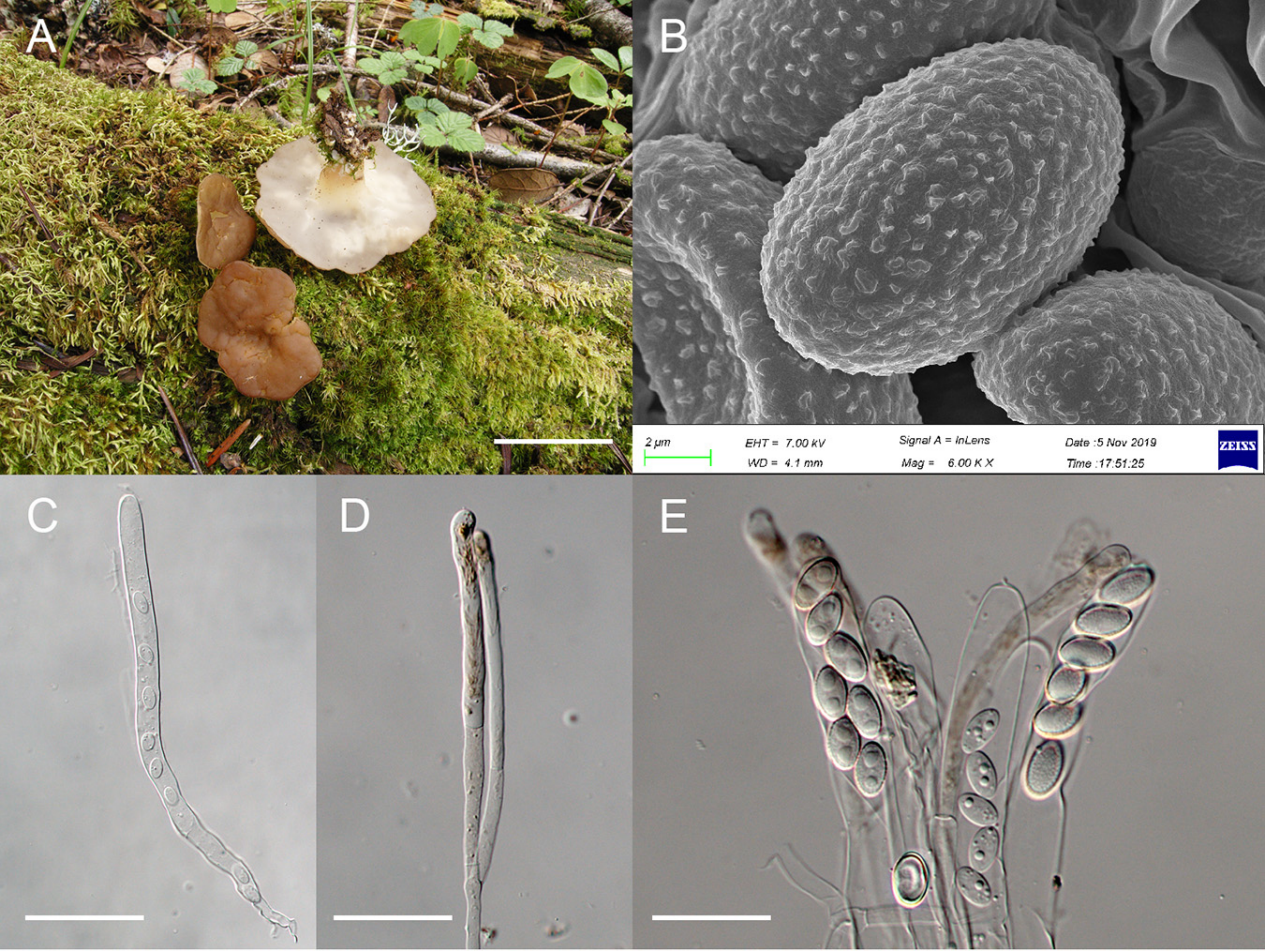
Pseudodiscina yunnanensis (HMAS 255832). (A) Fresh apothecia on nature substrate. (B) Ascospores under scanning electron microscope. (C) Immature ascus. (D) Paraphyses. (E) Ascospores within asci. Bars = 2.5 cm (A), 2 μm (B), 60 μm (C), 40 μm (D), and 30 μm (E).

**Pseudodiscina yunnanensis** X.C. Wang, Zhu L. Yang & W.Y. Zhuang, sp. nov. (Fig. 7).

Fungal Names FN571039

Etymology: The specific epithet refers to the type locality.

Typification: China, Yunnan Province, Lijiang City, Yulong Naxi Autonomous County, Yulong Snow Mountain, Yunshanping (grassland surround by forest of Picea sp.), 27°8′24″N, 100°13′55″E, alt. 3,200 m, on rotten wood, 13 August 2019, Zhu-Liang Yang 6275, HMAS 255832, holotype.

Apothecia discoid with margin reflex, stipitate, 2 to 3.5 cm in diameter when fresh, 1.5 to 2.5 cm in diameter and 1 to 1.5 cm high when dry; hymenium light brown to cinnamon when fresh, dark brown to blackish when dry, surface undulate-rugose; receptacle surface white to grayish white when fresh, whitish to buff when dry, glabrous; stipe subcylindrical, enlarged at upper portion, typically fluted with ribs, whitish to light brown, glabrous, internally hollow, 1 to 2 × 0.5 to 1 cm when fresh. Excipulum of textura intricata, hyphae hyaline, 9 to 16 μm wide. Asci subcylindrical, tapering toward the base, eight-spored, 210 to 245 × 13 to 17 μm. Ascospores ellipsoidal, hyaline, with fine markings on surface, biguttulate, nonapiculate, 13 to 16.5 × 8 to 10 μm. Paraphyses filiform, bifurcate, septate, hyaline, with brown contents in apical cells, 8 to 10.5 μm wide at apex, 5 to 6.5 μm below.

Notes: This species is similar to the type species of the genus in gross morphology (15, 35), but differs in smaller apothecia (2 to 3.5 cm in diameter versus up to 10 cm in diameter when fresh) with a convex hymenium surface instead of raised margin, and longer ascospores (13 to 16.5 μm versus 10 to 13 μm long). In addition, sequence divergences of the LSU region are 15 of 868 bp between this fungus and the type species of the genus.

**Pseudorhizina** Jacz., Opredelitel' Gribov. Sovershennye Griby (Diploidnye Stadii). I. Fikomitsety: 414, 1913.

= Helvellella S. Imai, Bot. Mag., Tokyo 46: 174, 1932.

= Ochromitra Velen., Monogr. Discom. Bohem.: 391, 1934.

= Gyromitrodes Vassilkov, Sovetsk. Bot. 6: 51, 1942.

Type species: Pseudorhizina korshinskii Jacz. (= Pseudorhizina sphaerospora (Peck) Pouzar).

Ascomata saddle-shaped or irregularly lobed, distinctly stipitate; hymenium grayish brown, dark brown to blackish when fresh; stipe deeply ribbed; receptacle surface pubescent. Asci operculate, commonly eight-spored, subcylindrical. Ascospores globose to ellipsoidal, nonapiculate, smooth, with one or two oil guttules.

Notes: Pseudorhizina was first synonymized with Gyromitra by Harmaja (12), later recognized as a valid generic name by him (25), and then further raised to a family level as *Pseudorhizinaceae* (24). Differently, Methven et al. (22) treated Pseudorhizina as a subgenus of Gyromitra based on the LSU phylogeny. Our sequence analyses revealed that it should be a separate genus belonging to *Discinaceae*, neither a subgenus of Gyromitra nor representing a separate family. It is a sister of Gyromitra, Hydnotrya, and Paragyromitra (Fig. 1). Two species are currently accepted in this genus, and P. sphaerospora occurs in China (23).

**Pseudoverpa** (P.A. Moreau, Bellanger, & Loizides) X.C. Wang & W.Y. Zhuang, stat. & gen. nov.

Fungal Names FN571040

≡ Gyromitra subgen. Pseudoverpa P.A. Moreau et al., Persoonia 40: 287, 2018.

Type species: Gyromitra anthracobia Loizides, P.A. Moreau and Bellanger.

Ascomata campaniform or cerebriform, stipitate; hymenium rufescent, gray-brown, purple-brown, or black when fresh; stipe smooth, white, hollow. Asci operculate, commonly eight-spored, subcylindrical. Ascospores ellipsoidal, smooth, mostly biguttulate.

Notes: Gyromitra subgen. Pseudoverpa P.A. Moreau et al. was established for G. anthracobia, a single species from Cyprus (36). Its gross morphology is similar to that of Gyromitra and Paragyromitra. However, our analyses presented in Fig. 1 to 3 indicated that this particular species should be considered an independent genus.

**Pseudoverpa anthracobia** (Loizides, P.A. Moreau & Bellanger) X.C. Wang & W.Y. Zhuang, comb. nov.

Fungal Names FN571041

≡ Gyromitra anthracobia Loizides, P.A. Moreau & Bellanger, Persoonia 40: 287, 2018.

Notes: This species was found in burned forest and characterized by its campaniform or cerebriform ascomata with blackish hymenium (36).

## DISCUSSION

Based on the three-locus and individual gene phylogenies (Fig. 1 to 4), a revised classification of *Discinaceae* is proposed. Eight genera are accepted: the delimitation of hypogeous Hydnotrya was retained; the concept of Gyromitra was amended; three generic names, Discina, Paradiscina, and Pseudorhizina, were recovered; and three new genera (Paragyromitra, Pseudodiscina, and Pseudoverpa) were established. Among them, Paradiscina and Pseudoverpa are currently monotypic. Accordingly, nine new combinations belonging to four genera have been proposed, and the descriptions of two new species have been made in Paragyromitra and Pseudodiscina.

The generic concept of Gyromitra accepted here is in its narrow sense. Our phylogenetic analyses reveal that certain taxonomic characters formerly used to distinguish genera, such as apothecial gross morphology and ascospore guttulation, are no longer considered critical criteria in the taxonomy of this group, although they can still be valuable in species identification. The biguttulate ascospores are present in six genera (Gyromitra, Paradiscina, Paragyromitra, Pseudodiscina, Pseudorhizina, and Pseudoverpa). Inhabitations like hypogeous or epigeous features are useful to distinguish some of the groups. For accurate species identification of the family, it is important to take into account not only their morphological characteristics but also results of multiple gene analyses that should be emphasized.

The multiple-gene phylogeny (Fig. 1) of the family seemingly recognized a monophyletic group consisting of the following genera: Gyromitra, Hydnotrya, Paragyromitra, and Pseudorhizina; in which the hypogeous Hydnotrya and epigeous Gyromitra are sisters. The study has determined a close relationship between Discina and Pseudodiscina. However, more comprehensive sampling and inferences based on additional genes are required to elucidate the complete evolutionary history of *Discinaceae*.

There might be an alternative treatment to retain only a single genus, i.e., Gyromitra, in the family *Discinaceae*. We do not think it is acceptable since the generic name Hydnotrya (1846) is earlier than Gyromitra (1849) and has the priority. However, Hydnotrya is commonly accepted as a hypogeous genus, living underground, and with tuberous ascomata. It is certainly not suitable for those fungi of the group with discoid, cerebriform, or saddle-shaped apothecia and again not supported by our multigene phylogeny. Integrated studies on apothecial gross morphology, traditional concepts, and molecular evidences should be considered to establish a reasonable classification of this family. In the taxonomic system we proposed, all the genera appeared to be monophyletic clades and with distinct morphology.

Many species placed in this family lack reliable reference sequences. It is possible that a few of the new species proposed nowadays based only on sequence analyses might be synonyms of old names that do not have molecular data. This is a common problem faced by the current taxonomists. However, in most cases for disicinaceous macrofungi, a morphological species is found to be a species complex that needs to be sorted out when sequence data are available. Thus, detailed and carefully morphological and anatomical examinations of specimens combined with molecular data are required for species identification of the group.

## MATERIALS AND METHODS

### Fungal materials and morphological observations.

Collections of *Discinaceae* from China deposited in the following fungaria were re-examined: Herbarium Mycologicum Academiae Sinicae (HMAS, https://nmdc.cn/fungarium/), Herbarium of Cryptogams, Kunming Institute of Botany, Chinese Academy of Sciences (HKAS), Herbarium of the Microbiology Institute of Guangdong (HMIGD), and Herbarium of Mycological Institute of Jilin Agricultural University (HMJAU). Specimens recently collected from the Guizhou, Hubei, and Yunnan provinces were identified (Table 2). The methods used for morphological observations followed our previous study (23, 37).

**TABLE 2:** Fungal species and sequences used in phylogenetic analyses^*a*^

Family	Species	Voucher specimen^*b*^	Locality	GenBank accession no.			Reference
				ITS^*c*^	LSU	TEF	
*Discinaceae*	Discina ancilis (Pers.) Sacc. 1889	MES-3058 = FLAS-F-61938	USA		MK430992	MK873427	48
	Discina brunnea (Underw.) Raitv. 1970	NY 01293396	USA		KC751520		22
		NY 01797001	USA		KC751523		22
	Discina caroliniana (Bosc.) Eckblad 1968	NY 01797002	USA		KC751528		22
		MICH NSW6105	USA		KC751501		22
	Discina fastigiata (Krombh.) Svrček and J. Moravec 1972	HMAS 254603	France	MG846992	MG847003	MG847049	22
		HAI-D-077	Ukraine	JQ691488		KF019285	49
		ALV 17918, epitype	Czech		MK784816		50
	Discina gigas (Krombh.) Eckblad 1968	HMAS 254604	France	MG846996	MG847005	MG847051	23
		TUR-A 208088, epitype	Czech	MH938663	MH938309		28
	Discina khanspurensis (Jabeen and Khalid) X.C. Wang and W.Y. Zhuang, comb. nov.	LAH 35074, holotype	Pakistan	MF116159			26
	Discina korfii Raitv. 1970	CUP 28997, paratype	USA	MW075385			27
		NY 03817715	USA	MW075391	MW078427		27
	Discina leucoxantha Bres. 1882	HMAS 279665	Spain	MG846991	MG847020	MG847066	23
		ILLS00121418	Andorra	MW078428			27
		NY 01797014	USA		KC751516		22
	Discina montana (Harmaja) Ginns 1975	BPI 566707, isotype	USA	MW077452	MW077442		27
		DAOM 706057	Canada	MW077460	MW077446		27
	Discina perlata (Fr.) Fr. 1849	HMAS 254607	France	MG846993	MG847022	MG847068	23
		O 86276	Norway		KX008334		Miller, unpublished
	Discina pseudogigas (X.C. Wang and W.Y. Zhuang) X.C. Wang and W.Y. Zhuang, comb. nov.	HMAS 46539, holotype	China: Sichuan	MG846994	MG847023	MG847069	23
		HMJAU 2596	China: Heilongjiang	ON554781	ON527646		This study
	Discina ticiniana (Littini) X.C. Wang and W.Y. Zhuang, comb. nov.	TUR-A 208095, epitype	Italy	MH938672	MH938317		28
		LUG 14130, holotype of Gyromitra littiniana A. Riva	Italy	MW075392	MW078429		27
	Discina sp.	420526MF0212	China: Hubei	ON554780	ON527645	ON568152	This study
	Gyromitra antarctica Rehm 1899	MES-1298	Argentina	MH930384			Mujic and Smith, unpublished
		AM-AR15-004	Argentina	KY462274			51
	Gyromitra esculenta (Pers.) Fr. 1849	HMAS 254602, epitype	France	MG846990	MG847002	MG847048	23
		HMAS 39743	Bulgaria	MG846989	ON527647		23 This study
	Gyromitra cf. esculenta	11-5-BC3	USA	KM204670	KM204700	KM034921	52
	Gyromitra splendida Raitv. 1974	TAAM 046650 = KL398	Estonia	KX185090	KX185094		53
	Gyromitra tasmanica Berk. and Cooke 1878	JAC8261	New Zealand	MK432687			Cooper et al., unpublished
		JAC14690	New Zealand	MK432809			Cooper et al., unpublished
	Gyromitra venenata Hai J. Li et al. 2020	HMAS 51681	USA	MG846988	MG847001	MG847047	23
		HKAS 107322 = MHHNU 7671, holotype	China: Hunan	MK253752	MT421931		29
		HMAS 255833 = ZhangY01	China: Guizhou	ON554782	ON527648	ON568153	This study
	Hydnotrya badia L. Fan et al. 2018	BJTC FAN270, holotype	China: Yunnan	MH445399	MH445405	OP846120	30 This study
	Hydnotrya bailii Soehner 1959	997	Germany	GQ149465			54
		B278	Estonia	FN669207			55
	Hydnotrya brunneospora L. Fan et al. 2018	HMAS 97138, holotype	China: Jilin	MH445404			30
	Hydnotrya cerebriformis Harkn. 1899	BJTC FAN645	China	MH430535	MH430557		30
	Hydnotrya cubispora (E.A. Bessey and B.E. Thomps.) Gilkey 1939	K(M) 104976	UK	EU784273			56
		DHP 05-605	Canada		DQ200845	KC109209	20, 57
	Hydnotrya laojunshanensis Lin Li et al. 2013	L2425, holotype	China: Yunnan	KC878618			58
		LL21211_BMDLU	China	ON982580	ON982621	OP846121	Li and Wan, unpublished; this study
		LL21212_BMDLU	China	ON982593	ON982622	OP846122	Li and Wan, unpublished; this study
		LL21215_BMDLU	China	ON982594	ON982623	OP846123	Li and Wan, unpublished; this study
	Hydnotrya michaelis (E. Fisch.) Trappe 1975	K(M) 61643	UK	EU784275			56
		6463-307	Germany	HM146816			59
		SOMF 30345	Bulgaria		MW879528		60
	Hydnotrya nigricans L. Fan et al. 2018	BJTC FAN349, holotype	China: Sichuan	MH445400	MH445406		30
	Hydnotrya puberula L. Fan et al. 2018	BJTC FAN721, holotype	China: Yunnan	MH445401	MH445407		30
	Hydnotrya tulasnei (Berk.) Berk. and Broome 1846	450	Germany	GQ149456			54
		MK62	Lithuania	MT603163			Grebenc et al., unpublished
		O 185729	Norway		KX008350		Miller, unpublished
	Hydnotrya sp.	JT19085	USA	MN653044	MZ018865		61
		JT19176	USA	MN653030	MZ018866		61
	Paradiscina melaleuca (Bres.) Benedix 1969	HMAS 279666	France	MG846998	MG847021	MG847067	23
		MPU:JCD 114-80	France	MT273636	MT273648	MT274708	62
	Paragyromitra ambigua (P. Karst.) X.C. Wang and W.Y. Zhuang, comb. nov.	HKAS 61885	China: Heilongjiang	ON554783	ON527649	ON568154	This study
		TAAM 017493 = KL397, holotype of Gyromitra infula var. *apiculatispora*	Estonia		KX185093		53
		O 185400	Norway		KX008347		Miller, unpublished
	Paragyromitra infula (Schaeff.) X.C. Wang and W.Y. Zhuang, comb. nov.	HMAS 42541	China: Hubei		ON527650		This study
		HKAS 53601	China: Sichuan		ON527651	ON568155	This study
		HKAS 55288	China: Yunnan		ON527652	ON568156	This study
		HKAS 62086	China: Yunnan		ON527653	ON568157	This study
		HMIGD 17887	China: Tibet	ON554784	ON527654	ON568158	This study
		HMIGD 18877	China: Tibet	ON554785	ON527655	ON568159	This study
		HMIGD 20214	China: Tibet	ON554786	ON527656	ON568160	This study
		HMJAU 22229	Belarus	ON554787	ON527657	ON568161	This study
		HMJAU 22386	Belarus	ON554788	ON527658	ON568162	This study
		HMJAU 24065	China: Jilin	ON554789	ON527659	ON568163	This study
		HMJAU 27038	China: Inner Mongolia	ON554790	ON527660	ON568164	This study
	Paragyromitra liangii X.C. Wang and W.Y. Zhuang, sp. nov.	HKAS 62299, holotype	China: Yunnan	ON554791	ON527661	ON568165	This study
	Paragyromitra tianshanensis (X.C. Wang and W.Y. Zhuang) X.C. Wang and W.Y. Zhuang, comb. nov.	HMAS 86057, holotype	China: Xinjiang	MG846963	MG847024	MG847070	23
		HMAS 88281	China: Xinjiang	MG846965	ON527662		Reference 23; This study
	Paragyromitra xinjiangensis (J.Z. Cao et al.) X.C. Wang and W.Y. Zhuang, comb. nov.	HMAS 24212, paratype	China: Gansu	MG846967	ON527663		Reference 23; This study
		HMAS 29393	China: Jilin	MG846968	ON527664		Reference 23; This study
		HMAS 74622	China: Sichuan	MG846972	ON527665		Reference 23; This study
		HKAS 46122	China: Tibet		ON527666	ON568166	This study
		HKAS 57677	China: Yunnan	ON554792	ON527667	ON568167	This study
		HMJAU 5556	China: Xinjiang	ON554793	ON527668	ON568168	This study
	Pseudodiscina melaleucoides (Seaver) X.C. Wang and W.Y. Zhuang, comb. nov.	MICH NSW 4520	USA		KC751517		22
		OSC 100037 = AFTOL-ID 55	Unknown		AY544663		AFTOL
	Pseudodiscina yunnanensis X.C. Wang, Zhu L. Yang and W.Y. Zhuang, sp. nov.	HMAS 255832 (= Yang 6275)	China: Yunnan	ON554794	ON527669	ON568169	This study
	Pseudorhizina californica (W. Phillips) Harmaja 1973	OSC 97268	USA	EU837202			Gordon unpublished
		OSC 69668	USA	EU837203			Gordon unpublished
		OSC 100068 = AFTOL-ID 176	USA		AY544673	DQ471059	AFTOL; reference 63
	Pseudorhizina sphaerospora (Peck) Pouzar 1961	HMAS 73375	China: Tibet	MG846997	MG847027	MG847073	23
		QFB3131	Canada	GQ304943	GQ305045		64
	Pseudoverpa anthracobia (Loizides et al.) X.C. Wang and W.Y. Zhuang, comb. nov.	LIP 0001407, holotype	Cyprus	MH014751	MH014750		36
		ML71422V2	Cyprus	MH014752	MH014749		36
*Geomoriaceae*	Geomorium fuegianum Speg. 1922	CT-4268 = FLAS-F-62903	Argentina	KY462314	MK430983	MK873418	48, 51
	Geomorium geodon Kraisit. et al. 2020	MES-2362	Chile	MK430942	MK430979	MK873413	48
	Geomorium singeri (Gamundí and E. Horak) Kraisit. et al. 2020	MES-2390	Chile	MK430943	MK430975	MK873415	48
*Helvellaceae*	Balsamia guozigouensis (L. Fan and Y.Y. Xu) L. Fan and Y.Y. Xu 2020	HMAS 97107, holotype	China: Xinjiang	MH910337	MH910328		65
	Balsamia nigrans Harkn. 1899	FLAS-F-60811 = MES-3108	USA	MT156518		MK873424	48
	Balsamia platyspora Berk. 1844	TUR 206101	Finland		MK100252	MK113871	66
	Dissingia confusa (Harmaja) K. Hansen and X.H. Wang 2019	FV2016060901	France	ON622916	ON622510		Mycoseq, unpublished
		O-253268 = H437	Norway		MK100254	MK113873	66
	Dissingia crassitunicata (N.S. Weber) T. Schumach. and Skrede 2019	ACD0462	USA	OL756007	OL742451		Dirks, unpublished
	Dissingia leucomelaena (Pers.) K. Hansen and X.H. Wang 2019	KH.06.01	USA		KC012682	KC109207	57
	Helvella bachu Q. Zhao et al. 2016	HKAS 88105, holotype	China: Xinjiang	KU739791	KU739815	KU739842	67
	Helvella calycina Skrede et al. 2017	O-253255	Norway	MN656158	KY772915	KY772833	68, 69
	Helvella crispa (Scop.) Fr. 1822	HKAS 75434	Germany	JX462572	KR493479	KT254487	70, 71
	Helvella lacunosa Afzel. 1783	O-255761	Norway	MN656169	MN655855	MN689302	69
	Pindara terrestris Velen. 1934	KH.12.67	Sweden		MK100279	MK113889	66
	Wynnella subalpine Q. Zhao et al. 2016	HKAS 45750, holotype	China: Tibet	KX034101	KT581118		72
		HKAS 94928	China: Sichuan		MK100278	MK113888	66
*Morchellaceae*	Morchella angusticeps Peck 1887	ISC-426682	USA	MT373929	MK430988	MK873428	48, 61
	Morchella esculenta (L.) Pers. 1794	M19	Czech	JQ723092	GU551586	GU551537	73, 74
	Morchella rufobrunnea Guzmán and F. Tapia 1998	M447	Mexico	JQ723129	GU551617	GU551568	73, 74
	Morchella tomentosa M. Kuo 2008	M105	USA	JQ723016	GU551028	GU551195	73, 74
*Tuberaceae*	Tuber luyashanense L. Fan 2022	BJTC FAN1031, holotype	China: Shanxi	OM256769	OM366157	OM649637	75
	Tuber maculatum Vittad. 1831	BJTC FAN868	China: Shanxi	OM265274	OM366227	OM649634	75
	Tuber turmericum L. Fan 2015	BJTC FAN471	China: Yunnan	KT758835	OM366208	OM649613	75, 76

a AFTOL, Assembling the Fungal Tree of Life; ITS, internal transcribed spacer; LSU, large subunit ribosomal DNA; TEF, translation elongation factor.

b Full names of the fungaria or herbaria: BJTC, Herbarium, Capital Normal University, China; BPI, U.S. National Fungus Collections; CUP, Cornell Plant Pathology Herbarium; DAOM, Canadian National Mycological Herbarium; FLAS, University of Florida Herbarium; ILLS, Illinois Natural History Survey Herbarium; ISC, Iowa State University Herbarium; K, Royal Botanic Gardens, Kew; LAH, University of the Punjab Herbarium; LIP, Université de Lille Herbarium; MICH, University of Michigan Herbarium; MPU, Université de Montpellier Herbarium; NY, New York Botanical Garden Herbarium; O, University of Oslo Herbarium; OSC, Oregon State University Herbarium; TAAM, mycological herbarium of Estonian University of Life Sciences; TUR, University of Turku Herbarium.

c GenBank accession numbers in bold type indicate the newly generated sequences.

### DNA extraction, PCR amplification, and sequencing.

Well preserved specimens were selected for DNA extraction using a Plant Genomic DNA kit (DP305, Tiangen Biotech, Beijing, China). Portions of ITS, LSU, and TEF genes were amplified by PCR using the primer pairs ITS5 and ITS4 (38), LROR and LR5 (39), and EF1-983F and EF1-1567R (40), respectively. Products were sequenced on an ABI 3730 DNA sequencer (Applied Biosystems).

### Phylogenetic analyses.

Forward and reverse sequences newly generated in this study were assembled using Seqman version 7.1.0 (DNASTAR Inc., Madison, WI, USA). These sequences and those retrieved from GenBank are listed in Table 2. To construct phylogenetic trees, three single-gene data sets (ITS, LSU, and TEF) and one combined data set were compiled. Sequences were aligned using MAFFT version 7.221 (41) and then manually edited via BioEdit version 7.1.10 (42) and MEGA version 6.0.6 (43). Maximum-likelihood (ML) analyses were performed using RAxML-HPC2 (44) on XSEDE 8.2.12 on CIPRES Science Gateway version 3.3 (45) with the default GTRCAT model. Bayesian Inference (BI) analyses were performed with MrBayes version 3.2.5 (46). Appropriate nucleotide substitution models and parameters were determined by Modeltest version 3.7 (47). The consensus trees were viewed in FigTree version 1.3.1 (http://tree.bio.ed.ac.uk/software/figtree/). Species of *Geomoriaceae*, *Helvellaceae*, *Morchellaceae*, and *Tuberaceae* served as outgroup taxa.
